# Sulforaphane promotes natural killer cell-mediated anti-tumor immune responses partially via cGAS-STING pathway in classical Hodgkin lymphoma

**DOI:** 10.1038/s41375-025-02627-1

**Published:** 2025-04-28

**Authors:** Ioanna Xagoraris, Ying Yang, Erofili Bougka, Dora Trogrlic, Persa Xyderou, Konstantina Stathopoulou, Nikolas Herold, Andreas Lundqvist, George Z. Rassidakis

**Affiliations:** 1 Department of Oncology-Pathology, Karolinska Institute, Stockholm, Sweden; 2 Department of Pathology and Cancer Diagnostics, Karolinska University Hospital, Stockholm, Sweden; 3 Childhood Cancer Research Unit, Department of Women’s and Children’s Health, Karolinska Institute, Stockholm, Sweden; 4 Paediatric Oncology, Astrid Lindgren Children’s Hospital, Karolinska University Hospital, Stockholm, Sweden

**Keywords:** Hodgkin lymphoma, Translational research

**To The Editor:**

Classical Hodgkin lymphoma (cHL) is one of the most common lymphoma types with approximately 3 new cases per 100,000 population per year in the Western world. With the current treatment approaches of combined chemoradiotherapy, cHL can be cured in the majority of patients [1]; however, a proportion of patients succumb to the disease and the long-term treatment side effects remain a challenge for Hematologists and Oncologists. New therapeutic strategies, including drug-conjugated anti-CD30 antibodies as well as immunotherapy with PD-1 or PD-L1 inhibitors [2, 3], may further improve clinical outcomes but also reduce the toxicities and side effects of traditional chemotherapy.

A unique morphologic feature of cHL is the presence of neoplastic Hodgkin and Reed/ Sternberg (HRS) cells in an inflammatory background consisting of reactive lymphocytes, histiocytes, plasma cells, eosinophils, and stroma cells (i.e., fibroblasts), that are considered part of the disease process [4]. The tumor microenvironment (TME) plays a pivotal role in the cHL pathogenesis because of the multiple and complex interactions of HRS cells with the inflammatory cells through numerous cytokines and chemokines [5]. HRS cells of cHL may still express major histocompatibility complex (MHC) class II, enabling the T-cell receptor of CD4+ T-lymphocytes to bind directly to MHC class II on the membrane of HRS cells [4]. The significant contribution of the inflammatory reaction in the pathogenesis of cHL makes this disease an excellent candidate for immunotherapy. Thus, uncovering the therapeutic effects of novel immunomodulators may contribute to cHL therapy.

Normal cells need to distinguish between their own DNA that localises in the nucleus, and the genetic material from pathogens, such as microbes or viruses, that can be localised in the cytosol. In cancer cells, host DNA may be localised in the cytoplasm as well. As a protective mechanism, the cells normally respond to cytoplasmic DNA by activating an inflammatory response. Cyclic GMP-AMP (cGAMP) synthase (cGAS) is a cytosolic DNA sensor that activates innate immune responses through production of the second messenger cGAMP, which activates the adaptor protein stimulator of interferon genes (STING). The latter then activates transcription factors IRF3 and NF-κB via kinases TBK1 and IKK, respectively, which induce the expression of interferons (IFNs) and cytokines [6]. Since discovery of novel immunomodulators would be very relevant in cHL, the potential impact of the natural compound sulforaphane (SFN) on anti-tumor immune responses was investigated here.

First, we report that SFN significantly inhibits cell growth and viability of cultured HRS cells through both inhibition of cell cycle progression and induction of apoptosis (Fig. 1A and Supplementary Figs. 1 and 2). Based on previously published findings, these effects can be attributed to inhibition of certain oncogenic pathways, including PI3K-AKT, AMPK and MEK-ERK [7], or other yet unknown mechanisms. However, SFN does not seem to inhibit the PI3K-AKT pathway (not shown) despite the reported activity of PI3K-AKT pathway in cHL [8]. The effects of SFN on the cell cycle seem to be mediated through upregulation of the CDK inhibitors p21 and/or p27, and downregulation of Cyclin D2 in a concentration dependent manner. Induction of apoptosis is associated with downregulation of the anti-apoptotic BCL2, BCL-xL and c-FLIP (Supplementary Fig. 1C). In line with these results are findings from previous studies in other hematologic malignancies, such as precursor T- and B-acute lymphoblastic leukemia, acute myeloid leukemia [9], pleural effusion lymphoma [10] as well as several solid tumors. Furthermore, SFN inhibited SAMHD1 phosphorylation, likely restoring its activity on cell growth inhibition as reported in other tumors, such as breast cancer [11, 12].

**Fig. 1:**
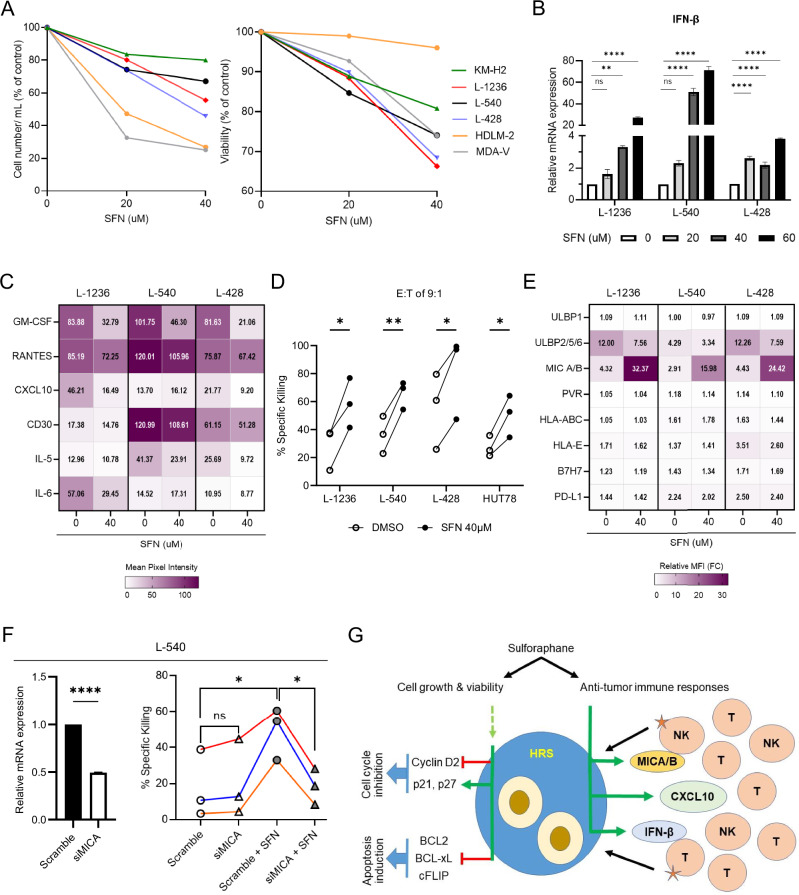
SFN inhibits cell growth and promotes natural killer cell-mediated anti-tumor immune responses and killing of cHL cells through activation of the cGAS-STING pathway. **A** Treatment of various cHL cell lines with increasing concentrations of SFN for 24 h resulted in decreased cell growth, shown as number of viable cells per mL of culture medium. Cell viability was also decreased at a concentration-dependent manner following SFN treatment for 24 h in cHL cells. The effects were variable among different cHL cell lines as shown. The cells were counted using trypan blue exclusion assay in triplicate. **B** Treatment with increasing concentrations of SFN for 24 h resulted in upregulation of IFN-β mRNA levels in all three cHL cell lines tested (L-1236, L-540, L-428) as assessed by reverse transcription-quantitative polymerase chain reaction (RT-qPCR) using GAPDH as the housekeeping gene. The experiment was repeated at least two times. Statistical differences were assessed by two-paired t-test (** *p* < 0.01; **** *p* < 0.0001; ns, not significant). **C** Using a human cytokine array (105 different cytokines, chemokines, growth factors and other soluble proteins) performed in cell culture supernatants at 24 h post SFN treatment at a concentration of 40 μM, GM-CSF, RANTES and angiogenin (Supplementary Table 1) protein levels were consistently decreased in all three cHL cell lines tested (L-1236, L-540, L-428). By contrast, MIF was increased in L-540 and L-428 cells, but not in L-1236 cells (Supplementary Table 1). Differential effects on the cytokine profile were observed among various cHL cell lines. The relative mean spot pixel intensity was determined by image analysis with ImageJ. Statistical differences were assessed by two-paired t-test (Supplementary Table 1). **D** NK cell killing assessed with Chromium 51 (^51^Cr) release assay was significantly increased at 4–6 h of co-culture following treatment with 40 μM of SFN for 24 h in all cHL cell lines assessed. HUT78, a T-cell lymphoma cell line (Sézary syndrome), was included for comparison (Supplementary Fig. 3A). More specifically, percentage of specific killing at 9:1 effector-to-target (*E*:*T*) ratio is shown. Statistical differences were assessed by two-paired t-test (* *p* < 0.05; ** *p* < 0.01). **E** Profiling of NK cell ligand expression on HL cell lines treated with 40 uM of SFN for 24 h. A dramatic increase of MICA/B and a decrease of ULBP2/5/6 expression (NK ligands) was observed. Other ligands assessed did not show significant differences following SFN treatment. The relative mean fluorescence intensity (MFI) was determined by flow cytometry (FC) and presented as fold change compared to unstained samples. Statistical differences were assessed by two-paired t-test (** *p* < 0.01; *** *p* < 0.001; **** *p* < 0.0001; ns, not significant). **F** To confirm the functional significance of the MICA/B ligands in mediating the NK cell killing, the ^51^Cr release assay was performed after MICA/B gene silencing with or without additional SFN treatment at a concentration of 40 μM for 24 h in L-540 cells. RT-qPCR (left panel) using GAPDH as the housekeeping gene confirmed adequate silencing of the MICA gene in L-540 cells. Similar results were obtained for L-428 cell line (Supplementary Fig. 4). Statistical differences were assessed by two-paired t-test (* *p* < 0.05; **** *p* < 0.0001). **G** The schematic summarizes the effects of SFN on cell growth and anti-tumor immune responses in cHL. (left) SFN seems to inhibit cell cycle progression through upregulation of the CDK inhibitors p21 and p27, and downregulation of Cyclin D2. These effects might be mediated by activation (dephosphorylation) of SAMHD1 or SAMHD1-independent pathways. Similarly, SFN seems to reduce apoptosis through downregulation of BCL2, BCL-xL (inhibitors of endogenous or mitochondrial apoptotic pathway) as well as cFLIP (inhibitor of exogenous or death-receptor apoptotic pathway). (right) In addition, SFN promotes anti-tumor immune responses through expression of IFN-β, CXCL10 and the NK cell-activating ligands MICA/B. These effects maybe mediated by activation of cGAS-STING pathway-dependent or -independent mechanisms. SFN sulforaphane.

In this report, we provide the first evidence that SFN promotes anti-tumor immune responses in cHL. At baseline, IFN-β, IFN-γ, and C-X-C motif chemokine ligand 10 (CXCL10) genes are differentially expressed among cHL cell lines tested (not shown). More specifically, SFN treatment modulates IFN-β gene expression (Fig. 1B), which is associated with activation of cGAS-STING pathway as shown by phosphorylation (activation) of TBK1 and its downstream target IRF3 (not shown). These effects also altered the cytokine profile in vitro (Fig. 1C, Supplementary Table 1). Among various chemokines and cytokines, granulocyte-macrophage colony-stimulating factor (GM-CSF), a hemopoietic growth factor and pro-inflammatory cytokine, showed the most consistently reduced production by HRS cells upon SFN treatment in all cHL cell lines assessed. It is likely that release of GM-CSF by HRS cells may alter the inflammatory TME in cHL, along with the production of GM-CSF by a variety of inflammatory cells, including T-cells, dendritic cells, macrophages, endothelial cells and fibroblasts, which are always present around the neoplastic HRS cells in the involved tissues. CXCL10 may show tumor proliferative or anti-proliferative effects depending on the expression status and subtype (A vs. B) of its receptor CXCR3 on the target immune cell. Although the magnitude of changes of IFN-β, CXCL10, and a variety of chemokines and cytokines following SFN treatment varies substantially among different cHL cell lines, they ultimately seem to contribute to anti-tumor immune responses as demonstrated by functional increase in natural killer (NK) cell-mediated killing of cHL cells in vitro (Fig. 1D). Interestingly, SFN treatment leads to a dramatic increase in the level of the NK cell-activating ligands MHC class I chain-related proteins A and B (MICA/B) [13] and to a lesser degree of other NK ligands in cHL, but not in control T-cell lymphoma cell line, HUT78 (Fig. 1E, Supplementary Fig. 3). To provide evidence for the functional significance of the MICA/B ligands in mediating the NK cell killing, SFN treatment after silencing of the MICA/B genes was performed, which significantly reduced the killing effect of SFN on co-cultured cHL cells (Fig. 1F, Supplementary Fig. 4). The critical role of MICA/B in mediating NK cell killing has been shown in other malignancies in vitro [14]. MICA/B are ligands recognised by the natural killer group 2, member D (NKG2D) receptor, which is expressed by NK cells, CD8+ Tcells and γδ T-cells. MICA/B are rarely expressed on the surface of healthy cells, but their expression is upregulated by DNA damage and cGAS-STING signaling, enabling recognition and elimination of infected and transformed cells by cytotoxic lymphocytes. It is tempting to speculate that high MICA/B production by the neoplastic HRS cells of cHL upon SFN treatment may promote anti-tumor immune responses through activation of the NK and CD8+ T- and NK cells in the TME resulting in tumor cell killing.

Since MICA/B expression is induced by the cGAS-STING signaling, we next investigated the cGAS-STING pathway as a mediator of immunomodulatory effects of SFN in cHL. STING is differentially expressed among cHL cell lines at the mRNA and protein level (not shown) in contrast to other lymphomas of B-cell origin [15]. As a first step, we demonstrated that the cGAS-STING pathway is functional in cHL cells since stimulation with a STING agonist resulted in increased IFN-β gene expression and CXCL10 protein production (Supplementary Fig. 5, Supplementary Table 2). To further elucidate the significance of this pathway, STING gene was silenced using specific siRNA constructs, which resulted in deactivation (dephosphorylation) of TBK1 and IRF3 (Supplementary Fig. 6). Knocking down STING also led to altered chemokine and cytokine profile, including decreased levels of CXCL10, interleukin (IL)-5, macrophage migration inhibitory factor (MIF), and notably CD30 (Supplementary Table 3). To further support the significance of the cGAS-STING pathway in anti-tumor immune responses in cHL, inhibition of STING activity using the selective inhibitor C-176 resulted in a dramatic decrease of CXCL10 gene expression, which was associated with altered cytokine and chemokine profile (Supplementary Fig. 7). These changes included a substantial decrease of thymus and activation-regulated chemokine (TARC) and an increase of the NK co-stimulator T-cell immunoglobulin and mucin domain 3 (TIM-3) levels (Supplementary Table 4). Notably, TARC serum levels represent an important biomarker for monitoring cHL [5] and has been associated with worse prognosis. TIM-3, a checkpoint receptor, is expressed by the HRS cells in a subset of cHL as well as in various immune cells of the TME. Previous studies have shown that blockade of TIM-3 in conjunction with PD-1 inhibition may suppress tumor progression in preclinical models and contribute to anti-tumor immune responses in several malignancies, thus supporting the development of TIM-3-based immunotherapeutic approaches [13].

In conclusion, we have shown that SFN is a suppressor of tumor growth and survival as well as a strong immunomodulatory agent that induces NK cell-associated anti-tumor immune responses in cHL, in part through STING-dependent mechanisms (Fig. 1G). The latter include expression and release of the NK cell-activating ligands MICA/B. These data suggest that pharmacologic modulation of the cGAS-STING pathway by SFN combined with other immunotherapy approaches and targeted therapy may contribute to novel investigational strategies in cHL.

## Supplementary information

Supplementary Material
