# Supplementary material for: Sulforaphane promotes natural killer cell-mediated anti-tumor immune responses partially via cGAS-STING pathway in classical Hodgkin lymphoma
Source: Leukemia. 2025 Apr 28;39(7):1787–90. doi: 10.1038/s41375-025-02627-1 (PMC12208876; doi:10.1038/s41375-025-02627-1)
Supplement: Supplementary file 1 — Supplementary Material [file 41375_2025_2627_MOESM1_ESM.docx]

**SUPPLEMENTARY MATERIAL**

**SUPPLEMENTARY FIGURES & LEGENDS**

## Suppl. Figure 1. SFN treatment results in inhibition of cell cycle and induction of apoptosis in cHL cells.

**A.** Cell cycle progression after SFN treatment with increasing concentrations for 24 hours was assessed by BrdU incorporation assay and flow cytometry analysis in L-540 cells. A concentration-dependent decrease in the S-phase fraction was observed suggesting inhibition of cell cycle progression and arrest at the G1-phase.

**B.** Apoptosis after SFN treatment with increasing concentrations for 24 hours was assessed by Annexin V staining and flow cytometry analysis in cHL cells. Increased apoptosis (Annexin V+ cells) at a concentration-dependent manner was seen in all three cHL cell lines tested (L-1236, L-540, L-428). Statistical differences were assessed by two-paired t-test (*, p<0.05; **, p<0.01; ***, p<0.001; ****, p<0.0001; ns, not significant).

**C.** The effects of increasing concentrations of SFN for 24 hours on cell cycle were associated with upregulation of the universal CDK inhibitor p27 in all three cHL cell lines assessed (L-1236, L-540, L-428) by Western blot and, additionally, with upregulation of another CDK inhibitor p21 in L-540 cells. Downregulation of Cyclin D2 was also observed in L-540 and L-428 cell lines. SAMHD1 phosphorylation was decreased as previously described [1]. The effects of SFN on apoptosis were linked with downregulation of the anti-apoptotic BCL2 and cFLIP in all three HL cell lines (L-1236, L-540, L-428). In addition, the anti-apoptotic BCL-xL was slightly downregulated in L-1236 and L-540 cells. β-Actin was used as the loading control. *SFN, Sulforaphane.*


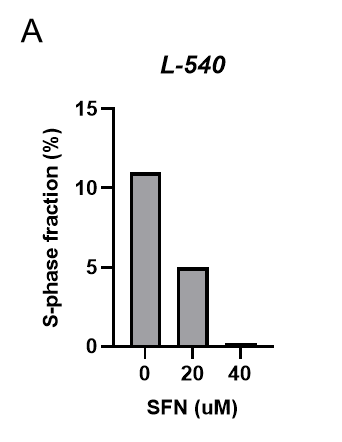

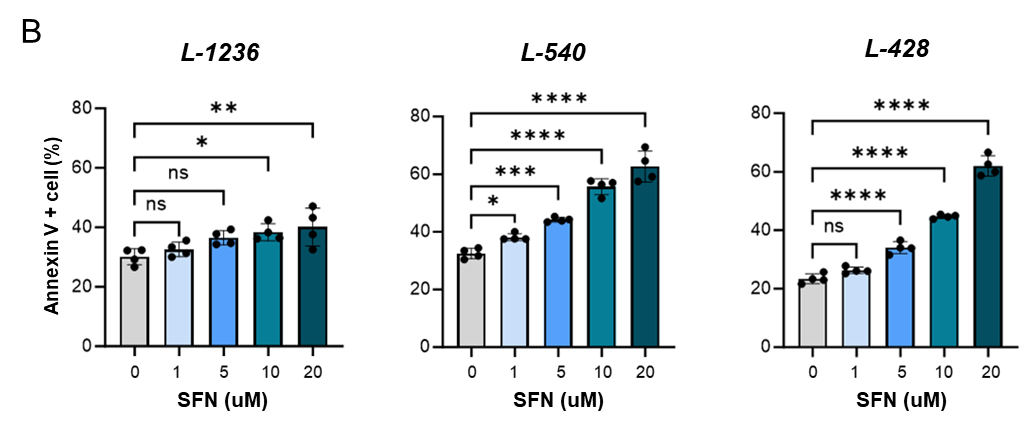


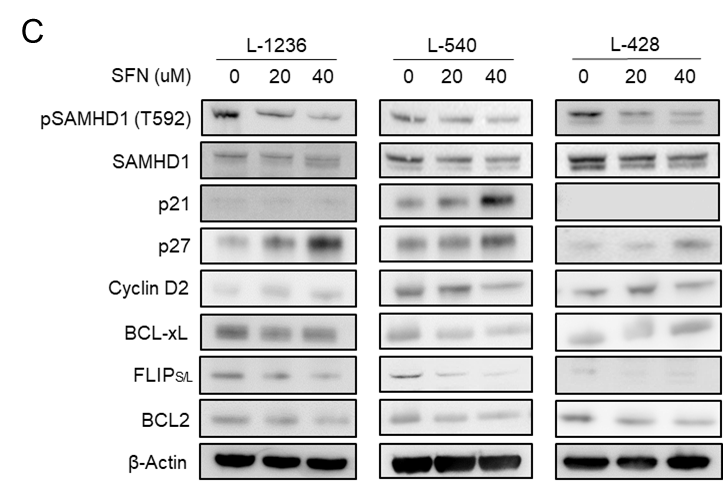


**Suppl. Figure 2. The effects of SFN on cell growth and viability in the T-cell lymphoma cell line HUT78.**


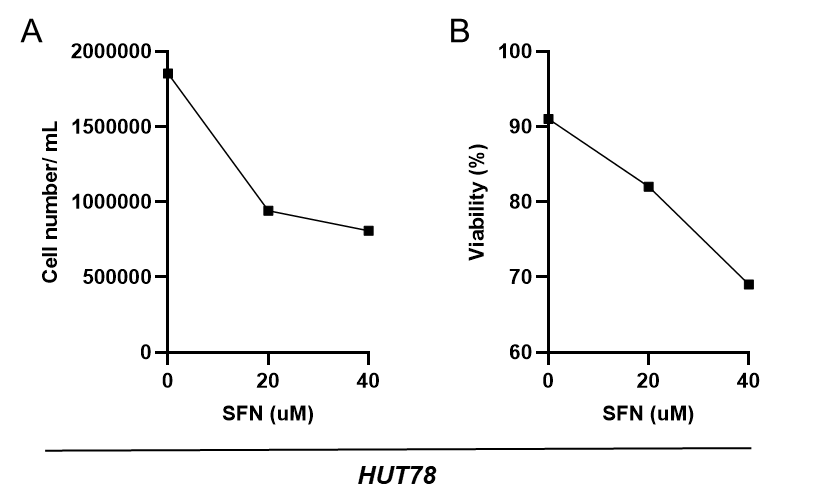
The effects of SFN on cell growth and viability were assessed in the T-cell lymphoma cell line HUT78. A concentration dependent decrease in both A) cell growth (cell number/ mL) and B) viability (%) was observed post 24 hours. The cells were counted using trypan blue exclusion assay in triplicate. *SFN, Sulforaphane.*

**Suppl. Figure 3.** **NK cell killing assay following SFN treatment in the T-cell lymphoma cell line HUT78.**

Treatment of HUT78 cells with increasing concentrations of SFN for 24 hours resulted in significantly increased NK cell killing at 4 and 6 hours using three different donors (A), which is associated with decreased levels of ULBP2/5/6 and PVR, but no changes in the MICA/B levels (B). These findings suggest that the effects of SFN on NK cell-mediated killing are not restricted to cHL. The relative mean fluorescence intensity (MFI) was determined by flow cytometry and presented as fold change compared to unstained samples. Statistical differences were assessed by two-paired t-test (*, p<0.05; **, p<0.01; ns, not significant). *SFN, Sulforaphane.*


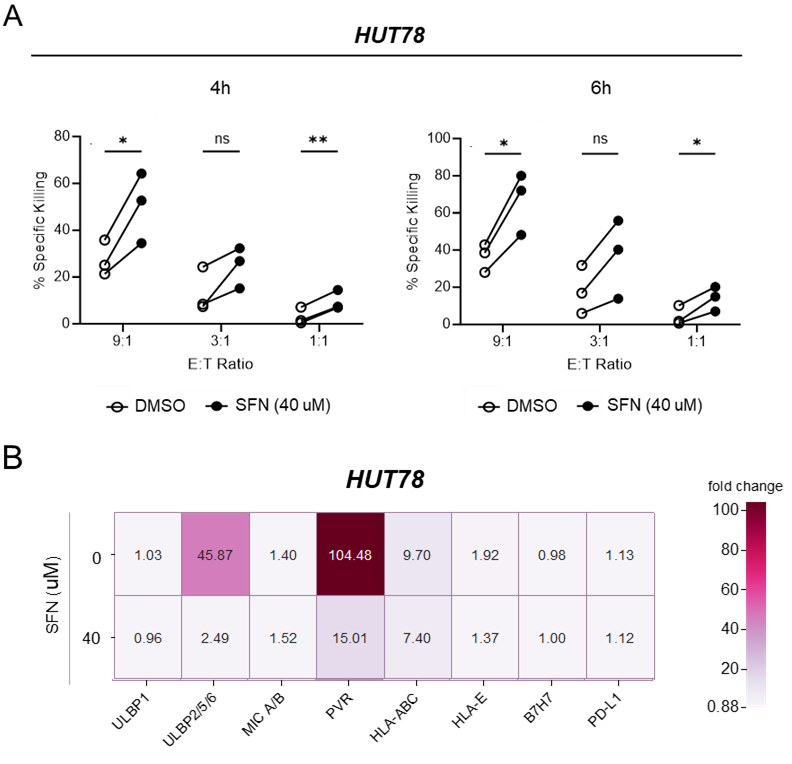


B

A

**Suppl. Figure 4. NK cell killing assay following MICA/B gene silencing in L-428 cells.**

Adequate knocking down of both MICA and MICB genes in L-428 cell line was confirmed by reverse transcription - quantitative polymerase chain reaction (RT-qPCR) at the mRNA level using GAPDH as the housekeeping gene (left panel). Simultaneous silencing of MICA and MICB genes resulted in significantly decreased NK cell killing at both 9:1 and 3:1 effector-to-target (E:T) ratios (right panel). Due to low viability, SFN treatment after MICA and MICB gene silencing could not be performed in L-428. Statistical differences were assessed by two-paired t-test (*, p<0.05; ****, p<0.0001).


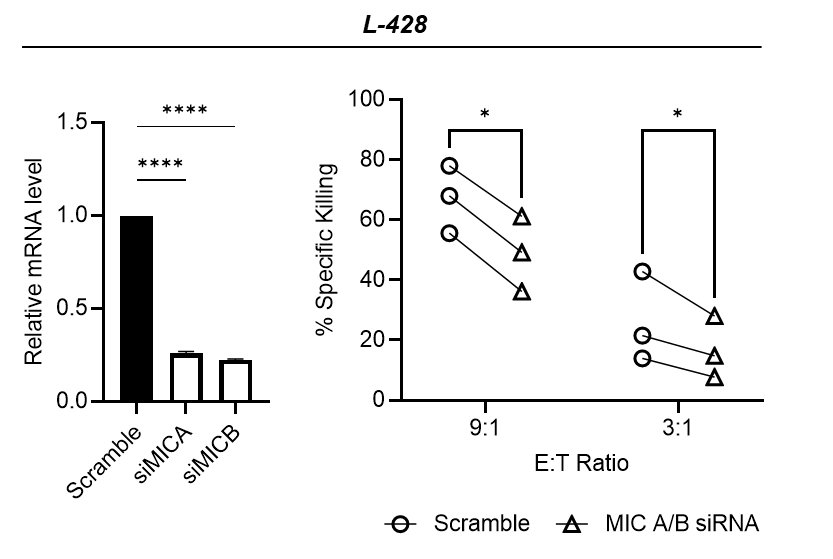


**Suppl. Figure 5. The effects of STING agonist on immune response mediators at the mRNA and protein level.**

1. Stimulation of the cGAS-STING pathway using a 2’3’-c-di-AM(PS)2 (STING agonist) at increasing concentrations for 48 hours resulted in increased IFN-β gene expression in all three cHL cell lines tested (L-1236, L-540, L-428), suggesting that the cGAS-STING pathway is functional and can be modulated in cHL cells. RT-qPCR data was normalized to the expression of GAPDH. The experiment was repeated at least two times. Statistical differences were assessed by two-paired t-test (***, p<0.001; ****, p<0.0001).
2. Stimulation of the cGAS-STING pathway with STING agonist at increasing concentrations for 48 hours resulted in its activation as shown by phosphorylation of STING, TBK1 and IRF3 in all three cHL cell lines tested (L-1236, L-540, L-428). β-Actin was used as the loading control. The experiment was repeated at least two times.
3. Cytokine profile of L-540 cells treated with 4 ug/mL of STING agonist for 48 hours using a cytokine array and cell culture supernatant. Only statistically significant differences are shown. Statistical differences were assessed by two-paired t-test (*, p<0.05; **, p<0.01; ***, p<0.001; ****, p<0.0001) (Suppl. Table 2).


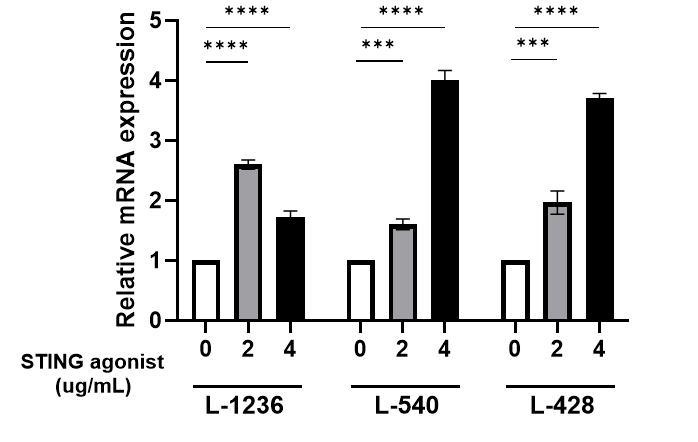

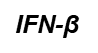


A

B


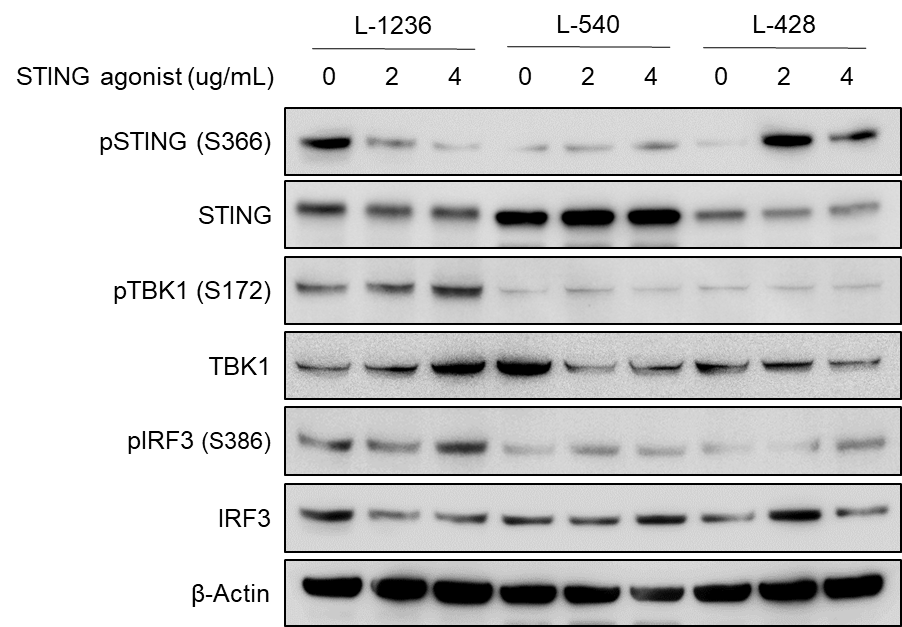


C


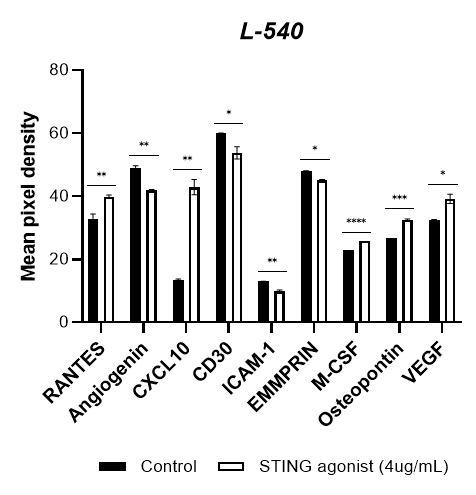

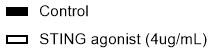


**Suppl. Figure 6. The effects of STING gene silencing in the immune response mediators in cHL cell lines.**

**A.** Adequate STING gene silencing using specific siRNA construct was confirmed at the mRNA level by RT-qPCR in all three cHL cell lines tested. RT-qPCR data was normalized to the expression of GAPDH. The experiment was repeated at least two times. Statistical differences were assessed by two-paired t-test (***, p<0.001; ****, p<0.0001).

**B.** Transient silencing of STING gene using specific siRNA construct resulted in decreased activation (phosphorylation) of TBK1 and/or IRF3 in all three cHL cell lines tested (L-1236, L-540, L-428). β-Actin was used as the loading control. The experiment was repeated at least two times.

**C**. Knocking down STING gene led to variable changes in the cytokine profile in L-540 cell culture supernatants post 72 hours. Only statistically significant differences are shown. The experiment was repeated at least two times. Statistical differences were assessed by two-paired t-test (**, p<0.01; ***, p<0.001; ****, p<0.0001) (Suppl. Table 3).


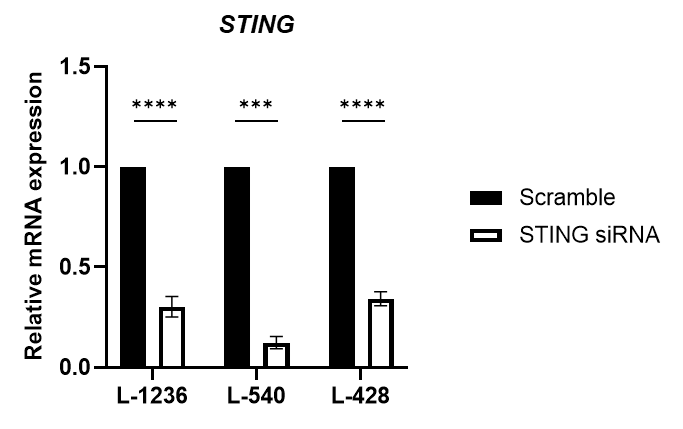

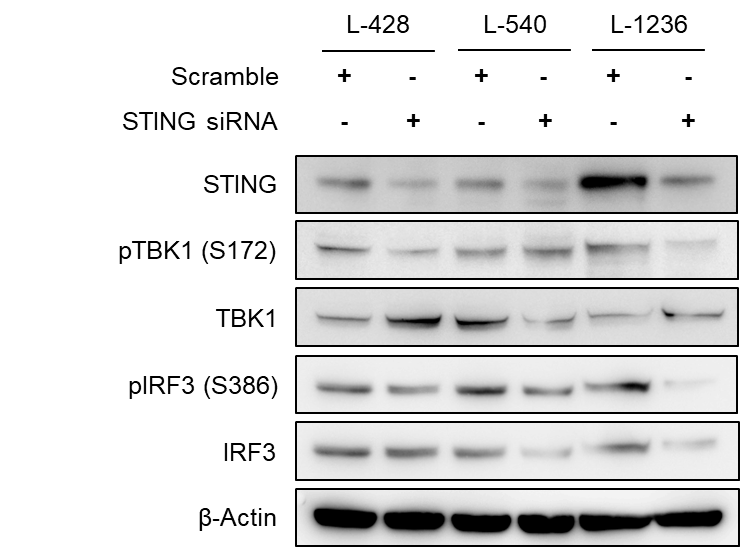


B

A

C

**Suppl. Figure 7. STING inhibition in L-540 cells.**

**A.** IFN-γ and CXCL10 gene expression was downregulated in L-540 cells using the selective STING inhibitor C-176 at increasing concentrations for 48 hours. RT-qPCR data was normalized to the expression of GAPDH. The experiment was repeated at least two times. Statistical differences were assessed by two-paired t-test (***, p<0.001; ****, p<0.0001).

**B.** Inhibition of STING activity by C-176 at increasing concentrations for 48 hours in L-540 cells resulted in increase of the NK co-stimulator T-cell immunoglobulin and mucin domain 3 (TIM-3), MIF and osteopontin as well as decreased levels of angiogenin, cystatin C, platelet-derived growth factor AA (PDGF-AA) and thymus and activation-regulated chemokine (TARC). Only statistically significant differences are shown. The two-paired t-test was used for all comparisons as shown (*, p<0.05; **, p<0.01) (Suppl. Table 4).

**
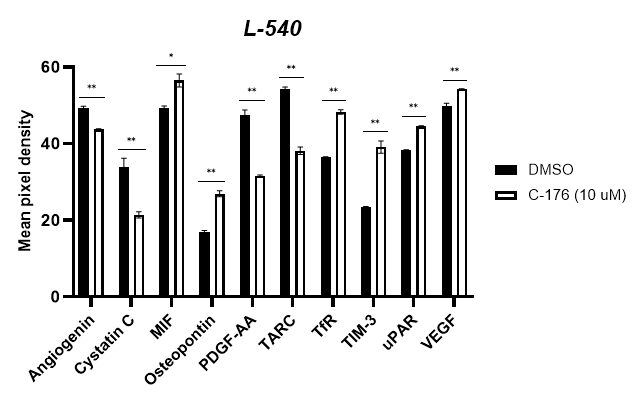
**

B

A

**SUPPLEMENTARY TABLES**

**Supplementary Table 1.** Differences in chemokine/cytokine expression after sulforaphane (40 uM) treatment in cHL cell lines.

- *L-1236*

| Protein | Mean Difference (+/-) | p-value |
| --- | --- | --- |
| Angiogenin | -15.70 | 0.000234 |
| CCL3/CCL4 (MIP-1a/MIP-1β) | -7.03 | 0.000007 |
| CXCL10 (IP-10) | -29.72 | 0.000003 |
| GM-CSF (CSF2) | -51.09 | 0.000001 |
| ICAM-1 (CD54) | -1.03 | 0.004988 |
| IL-6 | -27.61 | 0.000092 |
| MCP-1 (CCL2, MCAF) | -11.91 | 0.000182 |
| M-CSF (CSF1) | -6.82 | 0.001571 |
| MIF | -4.53 | 0.007518 |
| Osteopontin (OPN) | -18.18 | 0.000064 |
| PDGF-AA | -8.85 | 0.000001 |
| RANTES (CCL5) | -12.94 | 0.004003 |
| ST2 (IL-1 R4, IL1RL1, ST2L) | -11.78 | 0.000796 |
| TfR (CD71, TFR1, TFRC, TRFR) | -2.41 | 0.003848 |
| TNF-ɑ (TNFSF1A) | -10.84 | <0.000001 |
| VCAM-1 (CD106) | -16.42 | 0.000014 |
| VEGF (BEGFA) | +7.20 | 0.000279 |

- *L-540*

| Protein | Mean Difference (+/-) | p-value |
| --- | --- | --- |
| Angiogenin | -33.44 | 0.000263 |
| CD30 (TNFRSF8) | -12.39 | 0.000394 |
| DPPIV (CD26, DPP4, Dipeptidyl-peptidase IV) | +44.82 | 0.000046 |
| EMMPRIN (CD147, Basigin) | -6.07 | 0.002553 |
| GM-CSF (CSF2) | -55.45 | 0.000033 |
| ICAM-1 (CD54) | +8.54 | 0.000005 |
| IL-5 | -17.46 | 0.006888 |
| IL-17 (IL-17A, CTLAB) | -4.67 | 0.000371 |
| M-CSF (CSF1) | -18.21 | 0.000018 |
| MIF | +26.09 | 0.000060 |
| Osteopontin (OPN) | -11.21 | 0.012406 |
| PGF-AA | -34.84 | 0.000039 |
| RANTES (CCL5) | -14.05 | 0.000349 |
| TIM-3 (HAVCR2) | +9.35 | 0.000261 |
| TfR (CD71, TFRC, TRFR) | +12.92 | 0.000007 |
| uPAR (PLAUR) | +3.34 | 0.019795 |
| VCAM-1 (CD106) | +4.41 | 0.000212 |

- *L-428*

| Protein | Mean Difference (+/-) | p-value |
| --- | --- | --- |
| Angiogenin | -2.49 | 0.178361 |
| CD30 (TNFRSF8) | -9.88 | 0.000132 |
| CXCL10 (IP-10) | -12.57 | 0.000010 |
| EMMPRIN (CD147, Basigin) | -2.09 | 0.049288 |
| FGF-19 | -2.57 | 0.002841 |
| GM-CSF (CSF2) | -60.57 | 0.000003 |
| ICAM-1 (CD54) | -1.80 | 0.004357 |
| IL-5 | -15.97 | 0.000109 |
| M-CSF (CSF1) | -10.14 | 0.000037 |
| MIF | +1.06 | 0.130776 |
| Osteopontin (OPN) | -9.10 | 0.000088 |
| RANTES (CCL5) | -8.45 | 0.000152 |
| TARC (CCL17) | -8.73 | 0.015106 |
| VEGF (BEGFA) | -9.27 | 0.000001 |

**Supplementary Table 2.** Differences in chemokine/cytokine expression after STING agonist (4 ug/mL) treatment in L-540 cells.

| Protein | Mean Difference (+/-) | p-value |
| --- | --- | --- |
| Angiogenin | -7.12 | 0.001338 |
| CD30 (TNFRSF8) | -6.18 | 0.031665 |
| CXCL10 (IP-10) | +29.54 | 0.001611 |
| EMMPRIN (CD147, Basigin) | -2.90 | 0.011251 |
| ICAM-1 (CD54) | -3.21 | 0.006357 |
| M-CSF (CSF1) | +3.12 | 0.000094 |
| Osteopontin (OPN) | +5.87 | 0.000699 |
| RANTES (CCL5) | +6.99 | 0.005670 |
| VEGF (BEGFA) | +6.80 | 0.010703 |

**Supplementary Table 3.** Differences in chemokine/cytokine expression after STING gene silencing in L-540 cells.

| Protein | Mean Difference (+/-) | p-value |
| --- | --- | --- |
| CD30 (TNFRSF8) | -17.19 | 0.000295 |
| FGF-19 | -1.24 | 0.004569 |
| Flt-3 Ligand (FLT3LG) | -4.96 | 0.003952 |
| ICAM-1 (CD54) | -7.17 | 0.000009 |
| IL-5 | -5.51 | 0.000033 |
| IL-6 | -3.87 | 0.001242 |
| IP-10 (CXCL10) | -22.35 | 0.005830 |
| M-CSF (CSF1) | -6.23 | 0.000425 |
| MIF | -4.80 | 0.004216 |
| MMP-9 (CLG4B, Gelatinase B) | -4.73 | 0.000004 |
| Osteopontin (OPN) | -10.59 | 0.000485 |

**Supplementary Table 4.** Differences in chemokine/cytokine expression after STING inhibition with C-176 (10 uM) in L-540 cells.

| Protein | Mean Difference (+/-) | p-value |
| --- | --- | --- |
| Angiogenin | -5.63 | 0.004747 |
| Cystatin C (CST3, ARMD11) | -12.55 | 0.004588 |
| MIF | +7.10 | 0.010623 |
| Osteopontin (OPN) | +9.95 | 0.004391 |
| PDGF-AA | -15.80 | 0.003878 |
| TARC (CCL17) | -16.23 | 0.002455 |
| TfR (CD71, TFRC, TRFR) | +11.86 | 0.001269 |
| TIM-3 (HAVCR2) | +15.71 | 0.002575 |
| uPAR (PLAUR) | +6.26 | 0.001250 |
| VEGF (BEGFA) | +4.47 | 0.008840 |

**Supplementary Table 5.** List of cell lines used in the study.

| Cell Line | Lymphoma Type | Source |
| --- | --- | --- |
| L-1236 | Hodgkin lymphoma | DSMZ, Germany |
| L-540 | Hodgkin lymphoma | DSMZ, Germany |
| L-428 | Hodgkin lymphoma | DSMZ, Germany |
| KM-H2 | Hodgkin lymphoma | DSMZ, Germany |
| HDLM-2 | Hodgkin lymphoma | ATCC, USA |
| MDA-V | Hodgkin lymphoma | MDACC, USA* |
| MS | diffuse large B-cell lymphoma | DSMZ, Germany |
| HUT78 | cutaneous T-cell lymphoma | ATCC, USA |
| Mac2A | ALK-negative anaplastic large cell lymphoma | ATCC, USA |

* Kindly provided by Dr. R. J. Ford, The University of Texas MD Anderson Cancer Center, Houston, TX, USA.

**Supplementary Table 6.** Pharmacologic agents used in the study.

| Pharmacologic Agents | Catalog No. | Company |
| --- | --- | --- |
| R,S-Sulforaphane | S8044 | LKT Laboratories, Inc. |
| 2’3’-c-di-AM(PS)2 (Rp,Rp) | tlrl-nacda2r-01 | InvivoGen |
| IKKε/TBK1 Inhibitor II | 5.06306 | Sigma-Aldrich |
| C-176 | S6575 | Selleck Chemicals |

**Supplementary Table 7.** Antibodies used for Western blotting.

| Antibody | Clone | Catalog No. | Source |
| --- | --- | --- | --- |
| phospho-SAMHD1 | Thr592 | 89930 | Cell Signaling Technology |
| SAMHD1 |  | A303-691 | Bethyl Laboratories |
| phospho-STING | Ser366 | 19781 | Cell Signaling Technology |
| STING |  | 13647 | Cell Signaling Technology |
| phospho-TBK1/NAK | Ser172 | 5483 | Cell Signaling Technology |
| TBK1/NAK |  | 38066 | Cell Signaling Technology |
| phospho-IRF-3 | Ser386 | 37829 | Cell Signaling Technology |
| IRF-3 |  | 11904 | Cell Signaling Technology |
| phospho-IRF-7 | Ser477 | 12390 | Cell Signaling Technology |
| IRF-7 |  | 13014 | Cell Signaling Technology |
| NF-κB p65 |  | 8242 | Cell Signaling Technology |
| cGAS |  | 79978 | Cell Signaling Technology |
| phospho-AMPKα | Thr172 | 50081 | Cell Signaling Technology |
| phospho-AKT | Ser473 | 4051 | Cell Signaling Technology |
| p21 Waf1/Cip1 |  | 2946 | Cell Signaling Technology |
| p27 Kip1 |  | 3698 | Cell Signaling Technology |
| Cyclin D2 |  | sc-181 | Santa Cruz Biotechnology |
| FLIP_S/L_ |  | sc-8347 | Santa Cruz Biotechnology |
| BCL2 |  | M0887 | DAKO |
| BCL-xL |  | 2764 | Cell Signaling Technology |
| β-Actin |  | A5441 | Sigma-Aldrich |
| GAPDH |  | 97166 | Cell Signaling Technology |

**Suppl. Table 8.** Antibodies used for flow cytometry.

| Antibody | Catalog No. | Company |
| --- | --- | --- |
| Anti-Hu B7-H7 PE-Cyanine7 | 25-6537-42 | Invitrogen |
| FITC Mouse Anti-Human HLA-ABC | 560965 | BD Pharmingen |
| PE anti-human HLA-E | 342604 | BioLegend |
| APC anti-human MICA/MICB | 320908 | BioLegend |
| Brilliant Violet 785™ anti-human PD-L1 | 329736 | BioLegend |
| APC anti-human CD155 (PVR) | 337617 | BioLegend |
| Human ULBP-1 PerCP-conjugated Antibody | FAB1380C | R&D systems |
| Human ULBP-2/5/6 APC-conjugated Antibody | FAB1298A | R&D systems |

**Supplementary Table 9.** List of analytes represented in cytokine array.

| Adiponectin/Acrp30 | IFN-gamma | CCL2/MCP-1 |
| --- | --- | --- |
| Angiogenin | IGFBP-2 | CCL7/MCP-3 |
| Angiopoietin-1 | IGFBP-3 | M-CSF |
| Angiopoietin-2 | IL-1 alpha/IL-1F1 | MIF |
| Apolipoprotein A1 | IL-1 beta/IL-1F2 | CXCL9/MIG |
| BAFF/BLyS/TNFSF13B | IL-1ra/IL-1F3 | CCL3/CCL4  MIP-1 alpha/beta |
| BDNF | IL-2 | CCL20/MIP-3 alpha |
| CD14 | IL-3 | CCL19/MIP-3 beta |
| CD30 | IL-4 | MMP-9 |
| CD31/PECAM-1 | IL-5 | Myeloperoxidase |
| CD40 Ligand/TNFSF5 | IL-6 | Osteopontin (OPN) |
| Chitinase 3-like | IL-8 | PDGF-AA |
| Complement Component C5/C5a | IL-10 | PDGF-AB/BB |
| Complement Factor D | IL-11 | Pentraxin 3/TSF-14 |
| C-Reactive Protein/CRP | IL-12 p70 | CXCL4/PF4 |
| Cripto-1 | IL-13 | RAGE |
| Cystatin C | IL-15 | CCL5/RANTES |
| Dkk-1 | IL-16 | RBP4 |
| DPPIV/CD26 | IL-17A | Relaxin-2 |
| EGF | IL-18 BPa | Resistin |
| CXCL5/ENA-78 | IL-19 | CXCL12/SDF-1 alpha |
| Endoglin/CD105 | IL-22 | Serpin E1/PAI-1 |
| EMMPRIN | IL-23 | SHBG |
| Fas Ligand | IL-24 | ST2/IL1 R4 |
| FGF basic | IL-27 | CCL17/TARC |
| KGF/FGF-7 | IL-31 | TFF3 |
| FGF-19 | IL-32 alpha/beta/gamma | TfR |
| Flt-3 Ligand | IL-33 | TGF-alpha |
| G-CSF | IL-34 | Thrombospondin-1 |
| GDF-15 | CXCL10/IP-10 | TIM-1 |
| GM-CSF | CXCL11/I-TAC | TNF-alpha |
| CXCL1/GRO alpha | Kallikrein 3/PSA | uPAR |
| Growth Hormone (GH) | Leptin | VCAM-1 |
| HGF | LIF | VEGF |
| ICAM-1/CD54 | Lipocalin-2/NGAL | Vitamin D BP |

**SUPPLEMENTARY METHODS**

*Cell lines, pharmacologic agents and transfection experiments*

Six (6) cHL cell lines, L-1236, L-428, L-540, MDA-V, KM-H2, and HDLM-2 as well as control B- and T-cell lymphoma cell lines were used as listed in Suppl. Table 5. The pharmacologic agents used are shown in Suppl. Table 6. Cell viability was evaluated using trypan blue exclusion assay in triplicate. The cells were treated with SFN and other pharmacologic agents at various concentrations as shown in the corresponding figures. Transient transfection of cHL cell lines was performed as previously described [2] using specific siRNA constructs for MICA (cat. no. LQ-187896-00-0010, Horizon Discovery Ltd., Cambridge, UK), MICB (LQ-012178-00-0010, Horizon Discovery Ltd., Cambridge, UK), STING (cat. no. L-024333-00-0010, Horizon Discovery Ltd., Cambridge, UK), and IRF3 (cat. no. L-006875-00-0010, Horizon Discovery Ltd., Cambridge, UK). The Nucleofector I Device (Amaxa, Lonza, Basel, Switzerland) in combination with cell type-specific Nucleofector solutions and programs were utilized. Based on the viability and efficiency of transfection data provided by the manufacturer, the Nucleofector solution L (cat. no. VCA-1005, Lonza, Basel, Switzerland) and program X-01 was chosen for cell lines L-540 and KM-H2, and the Nucleofector solution V (cat. no. VCA-1003, Lonza, Basel, Switzerland) and program T-01 was chosen for cell lines L-1236 and L-428. Scrambled siRNA (cat. no. D-001810-10, Horizon Discovery Ltd., Cambridge, UK) was used as control in all sets of experiments.

*Gene expression analysis*

Part of the collected cells following treatment with SFN or other pharmacologic agents were used for gene expression analysis as described [3]. The housekeeping gene GAPDH (Thermo Fisher Scientific #Hs02786624_g1) was used in all sets of RT-qPCR reactions as a control (mRNA level of the gene of interest normalized to GAPDH mRNA).

*Western blot analysis*

Cells were collected during the exponential phase of growth in order to assess the baseline levels of various proteins, and at different time points in experimental settings as indicated. Cell pellets were washed twice with cold phosphate-buffered saline (PBS), and lysed for 30 min at 4°C in RIPA buffer containing protease and phosphatase inhibitors. Lysates were centrifuged at 14000 × g for 15 minutes to pellet the cell debris and collect the supernatant. Total protein concentration for each cell lysate was determined using the Bradford assay. Western blot was performed using standard methods as reported previously [3]. The primary antibodies used for Western blotting are listed in Suppl. Table 7.

*Flow cytometry assays*

Apoptosis was assessed with Annexin V staining and flow cytometry. The S-phase fraction of the cell cycle was assessed by a bromodeoxyuridine (BrdU) incorporation assay using the eBioscience BrdU Staining Kit for Flow Cytometry FITC (cat. no. 8811-6600-42, Invitrogen, Thermo Fisher Scientific Inc., Waltham, MA, USA) according to the manufacturer’s recommended protocol. Briefly, 10^4^ cells per well were incubated with BrdU diluted 1:100 in 96-well plates for 1 to 1.5 hours at 37°C. A FITC-conjugated BU20A monoclonal antibody (cat. no. 11-5071-42, Invitrogen, Thermo Fisher Scientific Inc., Waltham, MA, USA) was used at a 1:200 dilution according to the manufacturer's recommended protocol. After appropriate washings, the cells were analysed using flow cytometry. The expression of NK ligands was assessed by flow cytometry and the antibodies used are listed in Suppl. Table 8.

*NK cell cytotoxicity assay*

NK cells from human peripheral blood samples were obtained from healthy donors following the isolation of peripheral blood mononuclear cells (Ficoll density gradient centrifugation, GE Healthcare) and magnetic bead selection (Miltenyi Biotec, Human NK cells isolation kit). The purity of NK cells was confirmed by flow cytometry, with a CD3-/CD56+ population of >95%. Following magnetic bead isolation, NK cells were cultured in X-Vivo 20 media (Lonza) supplemented with 10% heat-inactivated human AB serum and 100 IU/ml interleukin (IL)-2 (Proleukin). Following a 48 hour culture, the cytolytic activity of NK cells was assessed using a chromium-51 (^51^Cr) release assay. Tumor cells were harvested and labeled with ^51^Cr (Perkin Elmer) as target cells. These labeled target cells were then seeded into a 96-well V-bottom plate to coculture with NK cells at specific E:T ratios. After 4 or 6 hours incubation, 25 ul of the culture supernatants were carefully transferred onto LUMA plates (PerkinElmer). The radioactivity of the LUMA plate was subsequently detected using MicroBeta2 (PerkinElmer).

*Cytokine Array*

To assess the levels of cytokines, chemokines, growth factors and other soluble proteins (Suppl. Table 9), cell culture supernatants and a membrane-based sandwich immunoassay (Proteome Profiler Human XL Cytokine Array Kit, cat. no. ARY022b, R&D Systems, Bio-Techne Ireland Limited, Ireland) was used according to the manufacturer's instructions. Three of the cHL cell lines, L-1236, L-540, L-428, were untreated (DMSO) or treated with 40 uM of SFN for 24 hours. Briefly, after 24 hours incubation, 800 µL of cell culture supernatant from each condition was collected and added to a previously blocked membrane-based antibody array, and incubated overnight at 4°C. At 18 hours, the membranes were washed, incubated with a detection antibody cocktail for 1 hour, washed and further incubated with streptavidin conjugated to horseradish peroxidase for 30 minutes. Expression was visualized using chemiluminescent detection reagents and ImageQuant LAS 4000 mini biomolecular imager (GE Healthcare Life Sciences, United States). Quantitation of the density of the bands was performed using the Image J software (National Institutes of Health and the Laboratory for Optical and Computational Instrumentation, University of Wisconsin) in triplicate.

**REFERENCES**

1. Sharifi HJ, Paine DN, Fazzari VA, Tipple AF, Patterson E, de Noronha CMC. Sulforaphane Reduces SAMHD1 Phosphorylation To Protect Macrophages from HIV-1 Infection. J Virol. 2022;96(23):e0118722.

2. Atsaves V, Tsesmetzis N, Chioureas D, Kis L, Leventaki V, Drakos E, et al. PD-L1 is commonly expressed and transcriptionally regulated by STAT3 and MYC in ALK-negative anaplastic large-cell lymphoma. Leukemia. 2017;31(7):1633-7.

3. Xagoraris I, Farrajota Neves da Silva P, Kokaraki G, Stathopoulou K, Wahlin B, Österborg A, et al. Sting Is Commonly and Differentially Expressed in T- and Nk-Cell but Not B-Cell Non-Hodgkin Lymphomas. Cancers (Basel). 2022;14(5).
